# Interobserver reproducibility of the PRECISE scoring system for prostate MRI on active surveillance: results from a two-centre pilot study

**DOI:** 10.1007/s00330-019-06557-2

**Published:** 2019-12-16

**Authors:** Francesco Giganti, Martina Pecoraro, Vasilis Stavrinides, Armando Stabile, Stefano Cipollari, Alessandro Sciarra, Alex Kirkham, Clare Allen, Shonit Punwani, Mark Emberton, Carlo Catalano, Caroline M. Moore, Valeria Panebianco

**Affiliations:** 1 Department of Radiology, University College London Hospital NHS Foundation Trust, London, UK; 2 Division of Surgery & Interventional Science, University College London, 3rd Floor, Charles Bell House, 43-45 Foley St, London, W1W 7TS UK; 3 Department of Radiological Sciences, Oncology and Pathology, Sapienza University of Rome, Rome, Italy; 4 Department of Urology, University College London Hospital NHS Foundation Trust, London, UK; 5 Department of Urology and Division of Experimental Oncology, Vita-Salute San Raffaele University, Milan, Italy; 6 Department of Urology, Sapienza University of Rome, Rome, Italy; 7 Centre for Medical Imaging, University College London, London, UK

**Keywords:** Prostatic neoplasms, Diffusion magnetic resonance imaging, Molecular imaging

## Abstract

**Objectives:**

We aimed to determine the interobserver reproducibility of the Prostate Cancer Radiological Estimation of Change in Sequential Evaluation (PRECISE) criteria for magnetic resonance imaging in patients on active surveillance (AS) for prostate cancer (PCa) at two different academic centres.

**Methods:**

The PRECISE criteria score the likelihood of clinically significant change over time. The system is a 1-to-5 scale, where 1 or 2 implies regression of a previously visible lesion, 3 denotes stability and 4 or 5 indicates radiological progression. A retrospective analysis of 80 patients (40 from each centre) on AS with a biopsy-confirmed low- or intermediate-risk PCa (i.e. ≤ Gleason 3 + 4 and prostate-specific antigen ≤ 20 ng/ml) and ≥ 2 prostate MR scans was performed. Two blinded radiologists reported all scans independently and scored the likelihood of radiological change (PRECISE score) from the second scan onwards. Cohen’s *κ* coefficients and percent agreement were computed.

**Results:**

Agreement was substantial both at a per-patient and a per-scan level (*κ* = 0.71 and 0.61; percent agreement = 79% and 81%, respectively) for each PRECISE score. The agreement was superior (*κ* = 0.83 and 0.67; percent agreement = 90% and 91%, respectively) when the PRECISE scores were grouped according to the absence/presence of radiological progression (PRECISE 1–3 vs 4–5). Higher inter-reader agreement was observed for the scans performed at University College London (UCL) (*κ* = 0.81 vs 0.55 on a per-patient level and *κ* = 0.70 vs 0.48 on a per-scan level, respectively). The discrepancies between institutions were less evident for percent agreement (80% vs 78% and 86% vs 75%, respectively).

**Conclusions:**

Expert radiologists achieved substantial reproducibility for the PRECISE scoring system, especially when data were pooled together according to the absence/presence of radiological progression (PRECISE 1–3 vs 4–5).

**Key Points:**

*• Inter-reader agreement between two experienced prostate radiologists using the PRECISE criteria was substantial.*

• *The agreement was higher when the PRECISE scores were grouped according to the absence/presence of radiological progression (i.e. PRECISE 1–3 vs PRECISE 4 and 5).*

• *Higher inter-reader agreement was observed for the scans performed at UCL, but the discrepancies between institutions were less evident for percent agreement.*

## Introduction

In the last decade, active surveillance (AS) has been increasingly used in the management of patients with favourable-risk prostate cancer (PCa), with compliance rates of more than 80% [1]. The role of magnetic resonance imaging (MRI) in this setting has also expanded, and there is evidence that almost 90% of academic centres in the USA routinely perform prostate MRI [2]. Serial MRI during AS protocols has been fully incorporated in the UK National Institute for Health and Care Excellence (NICE) guidelines [3–6]. However, there is still a lack of consistency in how serial MRI data during AS for PCa should be acquired and reported, either for an individual patient or across different cohorts.

In order to address this issue, in 2016, the European School of Oncology convened an international task force of experts in radiology, urology and radiation oncology to make recommendations on MRI reporting during AS. After the 2-day meeting, the Prostate Cancer Radiological Estimation of Change in Sequential Evaluation (PRECISE) recommendations were outlined [7]. The PRECISE recommendations aimed to define the conduct and reporting of an individual MRI scan and for cohorts of patients with serial MRI scans during AS follow-up [7].

Using a 1-to-5 scale (PRECISE score) for the reporting of the likelihood of radiological progression, the panel created a reporting proforma (case report) that should be used for each patient and for each MR scan, in order to collect the data in a systematic manner. At present, the PRECISE recommendations have been assessed in a single-centre cohort where all patients were rebiopsied after MRI. It was shown that those patients with a PRECISE score of 1 or 2 (57/158 (36%)) would not have been disqualified from AS at follow-up biopsy (i.e. the PRECISE criteria could allow patients with MR stability to safely avoid biopsy). The discrimination between the absence and presence of AS disqualification using a PRECISE score was demonstrated with a ROC curve of 0.83 [8].

However, in a similar manner to Prostate Imaging Reporting and Data System (PI-RADS) and Likert scoring systems [9–14], formal investigations of the inter-reader reproducibility of the PRECISE criteria are also needed to confirm that such recommendations can be universally recognised and applied. Thus, we conducted this study at two academic institutions (University College London (UCL) and Sapienza) to investigate the interobserver reproducibility of the PRECISE recommendations between two experienced radiologists, using scans from different MR machines and patient cohorts.

## Materials and methods

In this two-centre, retrospective study, patient records and MR images were reviewed as part of an audit routinely performed for the internal evaluation of the AS service. The two radiologists involved in the study (one from each centre; VP and FG, with 11 and 7 years of experience in prostate MRI reporting, respectively) had been actively involved in the discussion and drafting of the PRECISE recommendations.

### Patients

Anonymised scans from eighty patients (40 from each centre) were randomly selected from a list of eligible patients who met the following criteria: (i) being on AS with biopsy-confirmed low- or intermediate-risk PCa according to local guidelines (i.e. ≤ Gleason 3 + 4 and prostate-specific antigen-PSA-≤ 20 ng/ml); (ii) MR lesions were considered positive if they were concordant with the histology result using the six-sectors scheme (i.e. right/left base, midgland and apex); and (iii) two or more serial prostate MR scans conducted between April 2006 and May 2019.

### MR imaging protocol

At UCL, three different scanners were used: two 1.5-T (Symphony or Avanto, Siemens) and one 3-T system (Achieva, Philips), with a pelvic phased-array coil. At Sapienza, all exams were performed on a 3-T scanner (Discovery MR750, GE Healthcare) using a 32-multichannel surface phased-array body coil, but in some of the earlier scans, an endorectal coil was also used.

The multiparametric protocols in both centres included T2-weighted (T2-WI), diffusion-weighted imaging (DWI) (*b* values, 0, 100, 500 and 1000 s/mm^2^, and long *b* sequence, 1400 s/mm^2^ for 1.5-T or 2000 s/mm^2^ for 3-T scanners) with apparent diffusion coefficient (ADC) map calculation, and dynamic contrast-enhanced (DCE) imaging, as per international guidelines [15–17].

### Image review and analysis

Before the beginning of the study, both readers were provided ten practice MR cases from the other centre for training purposes, in order to allow them to get familiar with the MR images and MR sequences from both institutions, as different MRI magnet strength and workstations from different vendors had been used. As per PRECISE recommendations, the two radiologists were privy only to PSA and initial biopsy results but blinded to the original MRI reports [7].

Both readers reported all scans independently. Each scan was scored according to PI-RADS v.2.1 guidelines [17]. From the second scan onwards, each radiologist assessed the likelihood of radiological change (i.e. PRECISE score) from the previous scan, considering any change in size (according to the maximum diameter) or conspicuity (on any MRI sequence) of the lesion (Table 1). It should be recalled that the panel of experts who drafted the PRECISE recommendations concluded that there is still no consensus regarding the most accurate definition of volume (i.e. single maximum diameter *vs* biaxial measurement of maximum diameters *vs* the ellipsoid formula *vs* planimetry) and that further studies investigating such aspect are needed [7].

**Table 1:** Assessment of likelihood of radiological progression on magnetic resonance imaging in patients on active surveillance (PRECISE score)

PRECISE score	Assessment of likelihood of radiological progression
1	Resolution of previous features suspicious on MRI
2	Reduction in volume and/or conspicuity of features suspicious for prostate cancer
3	Stable MRI appearance: no new focal/diffuse lesions
4	Increase in size and/or conspicuity of features suspicious for prostate cancer
5	Definite radiologic stage progression (ECE, SV involvement, LN involvement, metastasis)

*MRI* magnetic resonance imaging, *ECE* extracapsular extension, *SV* seminal vesicle, *LN* lymph node

In accordance with PI-RADS v.2.1 guidelines [17], the lesion diameters were measured on the ADC map for the peripheral zone and on T2-WI for the transition zone. If lesion measurement was difficult or compromised on these sequences, this was made on the sequence that showed the lesion best, and the sequence used for measurement was recorded. As per PRECISE recommendations [7], in the case of multifocal disease, the index lesion included in the analysis was the lesion with the highest PI-RADS score and with the highest volume.

On a per-patient analysis (i.e. overall PRECISE score) and on a per-scan analysis (i.e. a single PRECISE score for each follow-up scan), we applied the following specific interpretation to the PRECISE criteria, which was agreed in consensus by both radiologists before the beginning of the study:

(i) ‘PRECISE 3’ (i.e. stability): either a scan with a stable lesion over time or a persistent negative scan.
(ii) In case of only diffuse MRI changes in the prostate gland (as seen in prostatitis, for example), the MR scan was reported as ‘negative’ for the presence of focal lesions.
(iii) ‘PRECISE 4’ (i.e. progression): either a new focal lesion (scored as PI-RADS ≥ 3) in a previous negative scan or a lesion with more suspicious MRI features (volume or conspicuity) since the last scan.

Each reader recorded the PRECISE scores independently. After study completion and data analysis, the results were collated and then discussed by the two radiologists in consensus.

### Statistical assessment

Clinical and demographic data are reported using descriptive statistics. Continuous variables are summarised by median and interquartile ranges (IQR) and categorical data by frequencies and percentages.

Inter-reader agreement was evaluated by using two methods: the *percent agreement* and Cohen’s kappa with standard quadratic weighting (*κ*_w_) [18–20]. The *percent agreement* was defined as the total number of concordant readings divided by the total number of readings made.

Each PRECISE score is mutually exclusive (i.e. there cannot be any overlap between variables), but it should be noted that the implications of a PRECISE score of 1 or 2 are similar (i.e. these patients are more likely to be monitored by clinical examination, PSA testing and serial MRI) and the same concept applies to those patients with a PRECISE score of 4 or 5 (i.e. it is highly expected that they will receive a targeted biopsy or active treatment). In order to take such differences into account, we used standard quadratic weighting (*κ*_w_) according to the following formula: *ω*_푖_ = 1−$\frac{i^2}{{\left(k-1\right)}^2}$, where *i* is the difference between categories and *k* is the total number of categories.

*κ*_w_ coefficients were interpreted as follows: 0.01–020, slight agreement; 0.21–0.40, minimal agreement; 0.41–0.60, moderate agreement; 0.61–0.80, substantial agreement; 0.81–0.90, strong agreement and > 0.90, almost perfect agreement. Statistical analyses were performed using SPSS (IBM, version 25).

## Results

Each radiologist assessed 80 baseline scans using PI-RADS v. 2.1 guidelines and 179 follow-up scans using the PRECISE criteria. The median number of MR scans per patient was 3 (IQR, 2.25–5) at UCL and 2 (IQR, 2–3) at Sapienza. The median interval between the first and the last scan (in months) was 51 (IQR, 29–77) at UCL and 23 (IQR, 13–34) at Sapienza.

Table 2 shows the baseline and follow-up characteristics of the population. 43/80 patients (54%) had at least one additional biopsy, 9/43 (21%) showed cancer upgrade (i.e. Gleason score ≥ 3 + 4, according to baseline histology), and 7/9 (78%) had an overall PRECISE score ≥ 4.

**Table 2:** Descriptive statistics of the patients included in the study for each group

	UCL (*n* = 40)	Sapienza (*n* = 40)
Age (years)	63 (56–68)	65 (60–71)
PSA (ng/ml)	6.19 (4.15–8.81)	4.4 (2.14–6.57)
Prostate volume (cc)	43.64 (31.8–63.38)	48.5 (32–68)
PSA density at baseline	0.12 (0.08–0.18)	0.08 (0.06–0.14)
Gleason score at entry		
3 + 3	33 [82]	37 [92]
3 + 4	7 [18]	3 [8]
Biopsy type at entry		
Systematic	36 [90]	37 [92]
Systematic + targeted	0	2 [5]
Targeted alone	4 [10]	1 [3]
Number of MR scans (*n* = 259)	151 [58]	108 [42]
Outcome		
No treatment	28 [70]	37 [92]
Active treatment	12 [30]	3 [8]
Treatment		
Radical prostatectomy	3 [26]	1 [33]
EBRT	1 [8]	1 [33]
Focal therapy	7 [58]	NA
Hormones	1 [8]	1 [33]

Data are median and interquartile range (parentheses); percentages in brackets [%]. Data for prostate volume and PSA density were calculated using the values from the original report

*UCL* University College London, *PSA* prostate-specific antigen, *NA* not applicable, *MR* magnetic resonance, *EBRT* external beam radiotherapy

Table 3 shows the number of PRECISE cases on a per-patient and on a per-scan basis. More than three quarters of the scans were reported as PRECISE 3 and 4. 23/80 patients (29%) did not develop any visible lesion (i.e. persistent negative scan) for both readers. At present, 14/80 (35%) patients have received treatment (Table 2).

**Table 3:** Number of PRECISE cases on a per-patient and on a per-scan basis, for each reader in the two different cohorts and in the overall population

Per-patient						
	UCL (*n* = 40)		Sapienza (*n* = 40)		Overall (*n* = 80)	
	Reader 1	Reader 2	Reader 1	Reader 2	Reader 1	Reader 2
PRECISE 1	3 (7)	3 (7)	3 (7)	2 (3)	6 (8)	5 (5)
PRECISE 2	1 (3)	1 (3)	5 (12)	1 (3)	6 (8)	2 (2)
PRECISE 3	21 (53)	20 (50)	23 (58)	27 (67)	44 (55)	47 (59)
PRECISE 4	10 (25)	11 (27)	8 (20)	10 (27)	18 (22)	21 (28)
PRECISE 5	5 (12)	5 (13)	1 (3)	NA	6 (7)	5 (6)
Per-scan						
	UCL (*n* = 111)		Sapienza (*n* = 68)		Overall (*n* = 179)	
	Reader 1	Reader 2	Reader 1	Reader 2	Reader 1	Reader 2
PRECISE 1	4 (4)	6 (5)	4 (6)	3 (3)	8 (5)	9 (5)
PRECISE 2	1 (1)	1 (1)	6 (9)	1 (1)	7 (4)	2 (1)
PRECISE 3	83 (75)	85 (77)	46 (68)	52 (77)	129 (72)	137 (76)
PRECISE 4	19 (17)	14 (13)	11 (16)	12 (19)	30 (16)	26 (15)
PRECISE 5	4 (3)	5 (4)	1 (1)	NA	5 (3)	5 (3)

Percentages in parentheses (%). At UCL, three different scanners were used: two 1.5-T (Symphony or Avanto, Siemens) and one 3-T system (Achieva, Philips), with a pelvic phased-array coil. At Sapienza, all exams were performed on a 3-T scanner (Discovery MR750, GE Healthcare) using a 32-multichannel surface phased-array body coil, but in some of the earlier scans, an endorectal coil was also used

*UCL* University College London, *NA* not available

### PRECISE score agreement

Overall, inter-reader reproducibility by kappa of each single PRECISE score was substantial both at a per-patient and a per-scan level (*κ* = 0.71 and 0.61, respectively), with quite a higher specific agreement rate (63/80, 79% and 145/179, 81%, respectively) (Table 4).

**Table 4:** Inter-reader agreement

	PRECISE score (1 to 5)		PRECISE 1–3 vs PRECISE 4–5	
Per-patient				
	*κ* value	Percent agreement (%)	*κ* value	Percent agreement (%)
UCL (*n* = 40)	0.81 [0.49–1]	80	0.95 [0.86–1]	97
Sapienza (*n* = 40)	0.55 [0.07–1]	78	0.66 [0.42–0.88]	90
Overall (*n* = 80)	0.71 [0.37–1]	79	0.83 [0.71–0.94]	90
Per-scan				
	*κ* value	Percent agreement (%)	*κ* value	Percent agreement (%)
UCL (*n* = 111)	0.70 [0.31–1]	86	0.74 [0.61–0.87]	93
Sapienza (*n* = 68)	0.48 [0.07–0.89]	75	0.56 [0.35–0.77]	88
Overall (*n* = 179)	0.61 [0.30–0.93]	81	0.67 [0.56–0.79]	91

0.41–0.60, moderate agreement; 0.61–0.80, substantial agreement; 0.81–0.90, strong agreement and > 0.90 almost perfect agreement; interquartile ranges in brackets [IQR]. At UCL, three different scanners were used: two 1.5-T (Symphony or Avanto, Siemens) and one 3-T system (Achieva, Philips), with a pelvic phased-array coil. At La Sapienza, all exams were performed on a 3-T scanner (Discovery MR750, GE Healthcare) using a 32-multichannel surface phased-array body coil, but in some of the earlier scans, an endorectal coil was also used

*UCL* University College London

The agreement was even stronger (*κ* = 0.83 per-patient and 0.67 per-scan) when the PRECISE scores were grouped according to the presence of radiological progression (i.e. PRECISE 1, 2 and 3 vs PRECISE 4 and 5), with a very high specific agreement (72/80, 90% and 163/179, 91%, respectively) (Table 4).

A closer look at Table 4 reveals that the two radiologists demonstrated higher inter-reader agreement (both by *κ* statistics and percent agreement) for the scans performed at UCL (*κ* = 0.81 vs 0.55 per-patient and *κ* = 0.70 vs 0.48 per-scan), but this was less evident in terms of percent agreement (32/40, 80% vs 31/40, 78% and 95/111, 86% vs 51/68, 75%, respectively).

Table 5 shows the number of overall single and grouped PRECISE scores for each reader. There were 19 discordant cases: 8/19 (42%) from UCL and 11/19 (58%) from La Sapienza.

**Table 5:** Overall PRECISE scores (*n* = 80) as assessed by each reader

		Reader 1							Total
		PRECISE 1	PRECISE 2	PRECISE 3	PRECISE 4	PRECISE 5	PRECISE 1,2 and 3	PRECISE 4 and 5	
Reader 2	PRECISE 1	2	2	1	1	0	–	–	6
	PRECISE 2	1	0	4	1	0	–	–	6
	PRECISE 3	2	0	40	2	0	–	–	44
	PRECISE 4	0	0	2	15	1	–	–	18
	PRECISE 5	0	0	0	2	4	–	–	6
	PRECISE 1, 2 and 3	–	–	–	–	–	(52)	(4)	(56)
	PRECISE 4 and 5	–	–	–	–	–	(2)	(22)	(24)
Total		5	2	47	21	5	(54)	(26)	80 (80)

Data in parentheses show the results according to radiological regression or stability (PRECISE 1, 2 and 3) and radiological progression (PRECISE 4 and 5)

## Discussion

The concept of ‘radiological progression’ in patients on AS for PCa is still relatively new, and there is a strong need of studies that can help in defining what ‘progression’ on MRI really is.

We observed substantial reproducibility in the application of the PRECISE scoring system in the whole cohort of 80 patients on AS for PCa (*κ* = 0.71; percent agreement = 63/80, 79%) between two highly experienced prostate radiologists from two different centres. We note that this was much lower for one of the centres (Sapienza) (*κ* = 0.55 per-patient and *κ* = 0.48 per-scan) and much higher for the other centre (UCL), where it was *κ* = 0.81 per-patient and *κ* = 0.70 per-scan.

From a clinical perspective, the recommendation based on MRI to biopsy patients with PRECISE scores of 4 or above was consistent across the two radiologists with *κ* = 0.83 and a percent agreement of 72/80 (90%). So, whilst there was lack of agreement within PRECISE scores 1–3, this would be below the threshold for clinical recommendation for further biopsy.

It is important to recall that the application of the PRECISE scoring system is still scarce in literature. At present, only one study by Dieffenbacher and colleagues [8] reports that patients with a PRECISE score ≥ 3 on follow-up should be rebiopsied. Moreover, the concept of ‘radiological progression’ is not well-defined, as there are yet no volume or diameter thresholds that allow us to reliably distinguish between expected interscan variability (which can be considerable [21]) and true progression.

The level of reproducibility of the PRECISE scoring system found in our study compares favourably with that reported for other scoring systems. Rosenkrantz and colleagues reported a substantial agreement for PI-RADS v.2 (*κ* = 0.59 in the peripheral zone and *κ* = 0.51 in the transition zone for PI-RADS ≥ 4) [10], and similar results (*κ* = 0.67) have been reported in the arterial hyperenhancement for the diagnosis of hepatocellular carcinoma using a 1-to-5 scoring system [22].

Differently from the PI-RADS guidelines, which any radiologist without prior expertise in prostate MRI would feel more familiar with, the PRECISE recommendations are not a rigid scoring system (i.e. there are no systematic flowcharts that can assist the inexperienced radiologist in scoring the scan). This is a key aspect of our study, as the pre-existing expertise in prostate MRI of both readers was important to determine the maximal reproducibility in this pilot study.

**Fig. 1:**
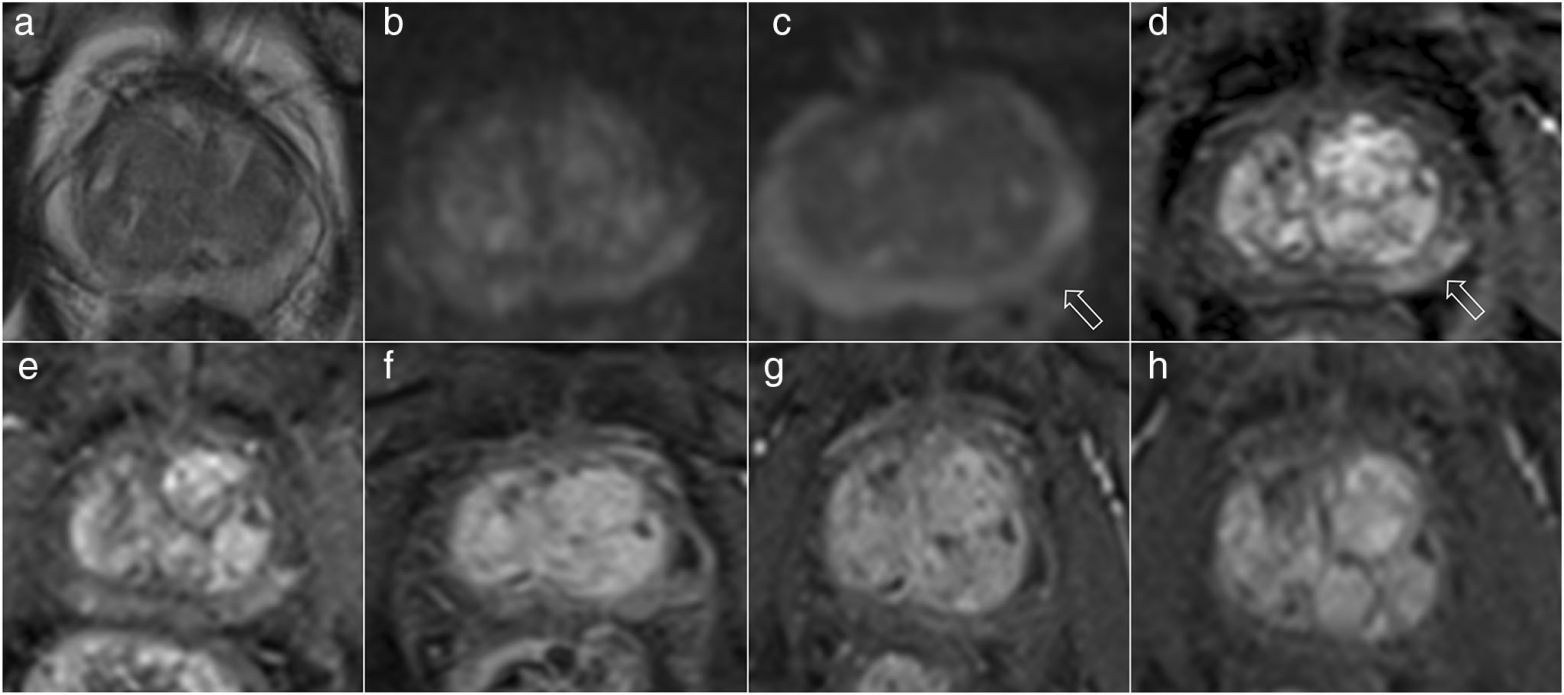
66-year-old patient on active surveillance for Gleason 3 + 3 (2 mm) in the left midgland peripheral zone on standard transrectal ultrasound biopsy and a presenting PSA of 13 ng/ml (PSA density, 0.17). The first 1.5-T MRI scan (**a**–**d**) shows a left-sided peripheral zone area (arrows) characterised by mild restricted diffusion on the ADC map (**c**) and early enhancement on dynamic contrast-enhanced imaging (**d**). The area showed stable MR appearance on dynamic contrast-enhanced imaging after one year (**e**) (scored as PRECISE 3 by both radiologists) and radiological regression (PRECISE 2) after one (**f**) and two years (**g**). The last scan (**h**) shows resolution of the previous suspicious MR features (PRECISE 1), with a PSA of 14 ng/ml and a PSA density of 0.16. The patient was discharged to his general practitioner for follow-up

**Fig. 2:**
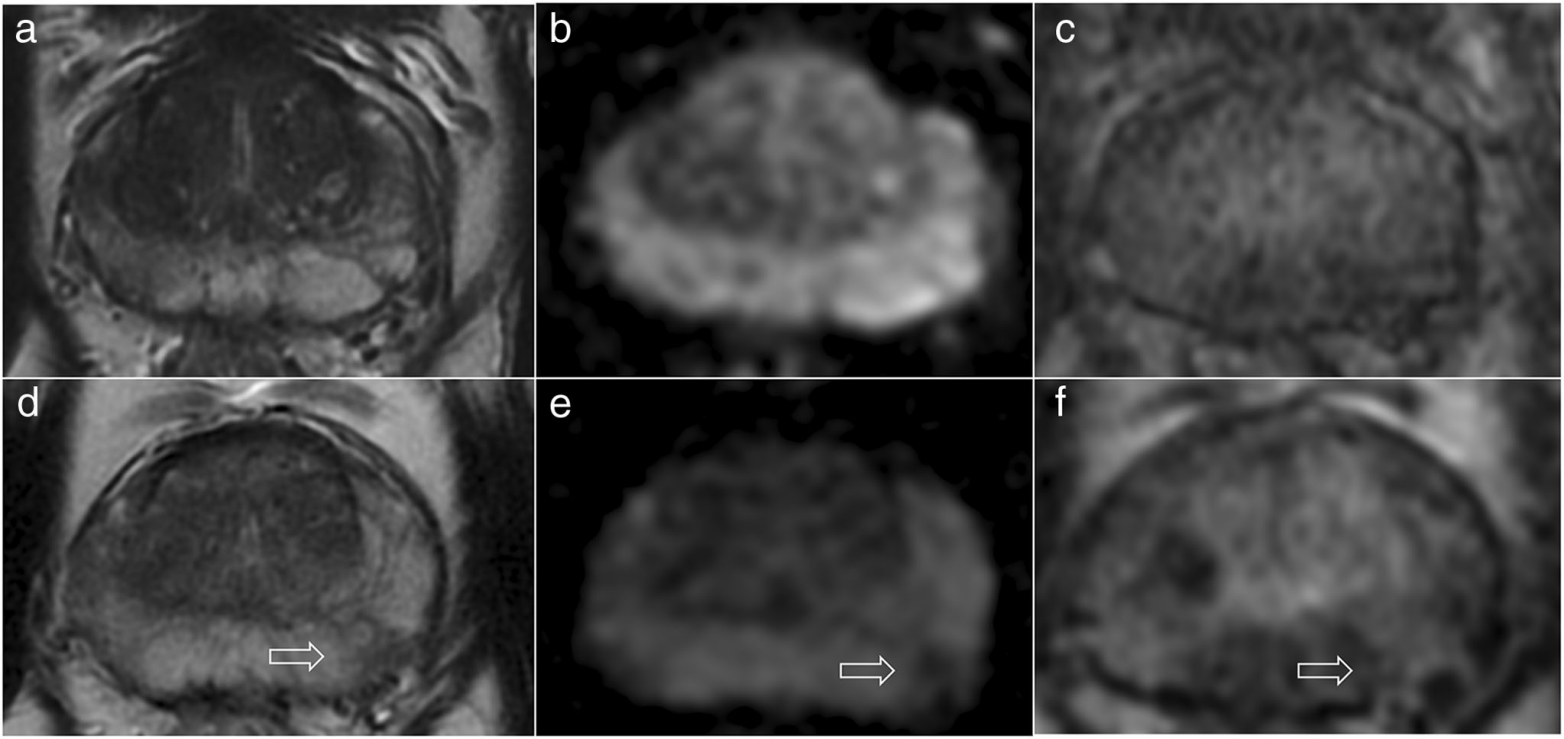
52-year-old patient on active surveillance for Gleason 3 + 3 (1 mm) in the right midgland peripheral zone and a presenting PSA of 6.02 ng/ml (PSA density, 0.12). The first 3-T MRI scan (**a**–**c**) did not show any focal lesion but only some patchy diffuse low T2-signal (**a**) and mild enhancement in the peripheral zone on the right (**c**) but no restricted diffusion on the ADC map (**b**). The scan after two years (**d**–**f**) revealed a new focal area (arrows) of low T2-signal (**d**), restricted diffusion on the ADC map (**e**) and mild enhancement (**f**) in the left peripheral zone, with a PSA of 8.89 ng/ml (PSA density, 0.18). The PRECISE score was 4 for both radiologists, and targeted biopsy of the area revealed Gleason 3 + 3 (3 mm)

*κ* coefficients and percent agreements were high in discriminating patients experiencing radiological progression, both on a per-patient and on a per-scan analysis (Figs. 1 and 2). These findings are of particular relevance as they suggest a strong agreement between two expert radiologists in identifying lesions showing radiological progression that should be targeted at biopsy, and suggest that MRI progression could be considered one of the drivers for triggering biopsies together with other clinical and laboratory findings [3, 23].

As shown in Table 4, the inter-reader agreement was higher for the scans performed at UCL (*κ* = 0.81 vs 0.55 per-patient; *κ* = 0.70 vs 0.48 per-scan), but this was less evident in terms of percent agreement (32/40, 80% vs 31/40, 78% and 95/111, 86% vs 51/68, 75%, respectively). A possible explanation is that 17/26 (65%) of the persistent negative scans (PRECISE 3) were from UCL and only 9/26 (35%) were from Sapienza, and the main reason lies in the early inclusion of MRI in the management of PCa in the UCL cohort. We know that in our study (i) the scans had been chosen at random from the database (i.e. there was no selection bias) and (ii) both radiologists had received a training set of scans from the other institution before commencing the study (i.e. no difference in MR reading confidence). Therefore, we believe that this difference could be mainly related to the small sample size of our study and also to the higher likelihood of inter-reader agreement for negative MR scans, as it has been previously shown that the mean number of lesions assigned per patient does not differ between different radiologists [24].

Our study has some limitations that should be acknowledged. First is that only two highly experienced radiologists assessed the PRECISE score, whilst in the aforementioned studies [10, 22], several radiologists with different levels of expertise had been involved. However, as the PRECISE scoring system has yet to be validated on a larger scale and there is still no consensus on how to define radiological progression, the contribution of two experts in the field provides a first answer to this. Further research will be required to evaluate the learning curve for inexperienced radiologists in reporting serial prostate MR scans and assessing a PRECISE score.

Second, this study involves two academic centres highly experienced in prostate MRI but with different MR systems and vendors. Whilst this could be seen as a limitation at a first glance, we believe that it could be considered a strength of this pilot study. It is known that readers from a single centre might approach the MR scans similarly, with a greater familiarity with the local imaging protocol, and this could result in greater inter-reader agreement. For this study, we provided each reader with a small initial set of MR scans to get familiar with the different MR systems in order to remove this potential bias.

Third, as this is a retrospective analysis of patients entering AS for clinical suspicion of PCa, the entry biopsy was often random, without a clear definition of the lesion location. Not all patients underwent rebiopsy during follow-up, and targeted resampling was often triggered by apparent radiological progression on MRI.

However, we believe that our findings could be useful for guiding future updates of the PRECISE criteria. The widespread use of the PRECISE recommendations could assist the radiological and urological communities in the identification of those patients on AS with radiological progression (i.e. PRECISE 4 and 5) so that rebiopsy or treatment could be delivered in a timely manner. At the same time, those patients with radiological regression or stability (i.e. PRECISE 1, 2 and 3) could avoid repeat biopsy, reducing the costs for the individual healthcare system.

## Conclusions

In conclusion, two experts achieved substantial reproducibility by using the PRECISE recommendations in two different academic centres. Overall, concordance between readers was highest in discriminating between radiological regression/stability (PRECISE 1–3) and progression (PRECISE 4 and 5).

## Abbreviations

AS: Active surveillance
DCE: Dynamic contrast enhanced
DWI: Diffusion-weighted imaging
MRI: Magnetic resonance imaging
NICE: National Institute for Health and Care Excellence
PCa: Prostate cancer
PI-RADS: Prostate Imaging Reporting and Data System
PRECISE: Prostate Cancer Radiological Estimation of Change in Sequential Evaluation
PSA: Prostate-specific antigen
T2-WI: T2-weighted imaging
TRUS: Transrectal ultrasound
UCL: University College London
